# The Multiple Role of Silicon Nutrition in Alleviating Environmental Stresses in Sustainable Crop Production

**DOI:** 10.3390/plants11091223

**Published:** 2022-04-30

**Authors:** Szilvia Kovács, Erika Kutasy, József Csajbók

**Affiliations:** Institute of Crop Sciences, Faculty of Agricultural and Food Sciences and Environmental Management, University of Debrecen, Böszörményi Str. 138, H-4032 Debrecen, Hungary; szkovacs@agr.unideb.hu (S.K.); csj@agr.unideb.hu (J.C.)

**Keywords:** silicon, stress alleviation, plant nutrition, phytoliths

## Abstract

In addition to the application of macronutrients (N, P, K), there has been an increasing interest in studying the effects of different micronutrients on growth and development in plant populations under abiotic and biotic stresses. Experimental results have demonstrated the role of silicon in mitigating environmental stresses on plants (especially in silicon accumulating plant species). Furthermore, as the silicon content of soils available to plants can vary greatly depending on soil type, the many positive results have led to increased interest in silicon as a nutrient in sustainable agriculture over the last decade. The grouping of plant species according to silicon accumulation is constantly changing as a result of new findings. There are also many new research results on the formation of phytoliths and their role in the plants. The use of silicon as a nutrient is becoming more widespread in crop production practices based on research results reporting beneficial effects. Controversial results have also been obtained on the use of different Si-containing materials as fertilizers. Many questions remain to be clarified about the uptake, transport, and role of silicon in plant life processes, such as stress management. Future research is needed to address these issues. This review discusses the role and beneficial effects of silicon in plants as a valuable tool for regulating biological and abiotic stresses. Our aim was to provide an overview of recent research on the role and importance of silicon in sustainable crop production and to highlight possible directions for further research.

## 1. Introduction

The role and significance of silicon (Si) in plants’ physiology has been recognized recently and it is classified as a beneficial plant nutrient in some of the most significant crops grown in the largest area.

Silicon is the second element after oxygen in abundance on the Earth; it is common in nearly all soils and available for plants. The visible symptoms of Si deficiency or toxicity are not striking, and it does not harm plants when accumulated in excess surplus [1]. Silicon research in plants have started more than a century ago, but until the beginning of the 20th century, its role and beneficial effects on crop production were not recognized [2]. 

Japanese scientists started experiments on the importance of silicon in the stability of rice production. One of the first reports on Si research published in a scientific journal of agronomy was written by Onodera [3]. He studied the chemical composition of rice plants collected from different regions of Japan and reported that the Si content of rice leaves infected with blast disease was always lower than that of healthy leaves originating from the same field. He found that the natural silicon content of rice tissues was different in the healthy plants that originated from different paddy fields. Although Si is not an essential element for plant growth, these results initiated intensive studies on the effects of silicon based on their achievements. Si was recognized as valuable fertilizer in crop production as it enhances the healthy growth and development of different crops and has a significant role in the resistance of plants to biotic and abiotic stresses by promoting several plant physiological processes. The application of silicon fertilization can reduce the usage of pesticides and fungicides. Thereby, Si is now considered an environment-friendly element [4,5].

## 2. Analysis of Silicon in Plants

There are several methods to determine total Si in plants. In general, all of the analytical methods involve two major steps. The first one is to dissolve Si contained in the insoluble silicates and extract or isolate Si from the materials, and the second one is to gauge Si based on gravimetric methods, spectrometric methods, or microscopic observation. X-ray fluorescence spectrometry (XRF) is a nondestructive technique for multi-elemental analysis of soil and plant materials showed even higher measurement accuracy for Si over the destructive methods based on alkaline fusion or acid. 

Gravimetric methods and spectrometric methods [6] were widely used to determine the total Si content of plant biomass. Within each group, there are many unique versions and protocols to choose from. In rice straw, the gravimetric method was used, during which organic matter was removed by acid digestion [7]. Snyder [8], after removing organic material by heat treatment (cremation), filters the ash with hydrochloric acid to remove excess elements. After filtration, the weight of the ash-coated filter paper is measured and, finally, the silicon is removed by hydrofluoric treatment. The difference in weight before and after the hydrofluoric (HF) treatment gave Si content. Before the use of spectrometric methods, special sample preparation protocols are required to recover the silicon from the plant sample, such as the lithium metaborate fusion method [9], autoclave-induced digestion method [10], hydrofluoric acid extraction method [11], and oven-induced digestion (OID) method [12]. After having the sample prepared, silicon can be determined by using light absorption spectrometer (using blue or yellow Si molybdenum method) [13], atomic absorption spectrometer (AAS) [14], inductively coupled plasma spectrometer (ICP) [15], and X-ray fluorescence spectrometer (P-XRF) [16].

## 3. Availability of Silicon for Plants

Datnoff and Rodrigues [5] pointed out that genetics plays an important role in Si accumulation. In the same silicon accumulating species, the varieties may differ in their silicon content and their reaction to Si fertilization. Although Si accumulation is a phylogenetic feature, the availability of Si will influence the amount of Si absorbed by plants. Most of the research on silicon application is carried out with the silicon accumulating crops. But, in addition, silicon fertilization has effects on other crops, also. Clarifying the role of Si in crop production and understanding the mechanisms that regulate Si uptake by plants in soil and in planta is important and justified research worldwide [17].

Silicon generally can be found in the earth’s crust and the soil contains 1 to 5% silicon in an active form. The form of silicon that is available to plants as a nutrient is called monosilicic acid (H_4_SiO_4_) and the amorphous silicon-dioxide form is also can be readily uptaken by plants. The availability and amount of this element are influenced by the properties of the soil, including pH, clay content, organic matter, iron (Fe) or aluminum (Al) oxides/hydroxides [18,19], and microbial activities. [20]. 

The silica content of the soil is slightly soluble and the dissolution (SiO_2_(s) + 2H_2_O = H_4_SiO_4_), is enhanced by organic acids in soils [21]. Bioweathering of silicates involving silicate-solubilizing bacteria is known to have a significant part in the solubilization of insoluble silicon due to the production of organic acids [22,23]. These beneficial microbes enhance the availability of silicon to plants under different stress conditions through better uptake of these minerals [23,24]. Different bacterial strains of genus *Bacillus*, *Pseudomonas*, *Proteus*, *Rhizobia*, *Burkholderia*, and *Enterobacter* have been found to release silicon from silicates and enhance plant growth [23,25,26,27,28,29,30]. Microbial weathering of asbestos (which are highly toxic silicate minerals due to the presence of high concentrations of the transition metal iron found in soil by bacteria) could be a detoxification process inhibiting asbestos toxicity [31].

## 4. Silicon Uptake and Transport in Plants

**Figure 1:**
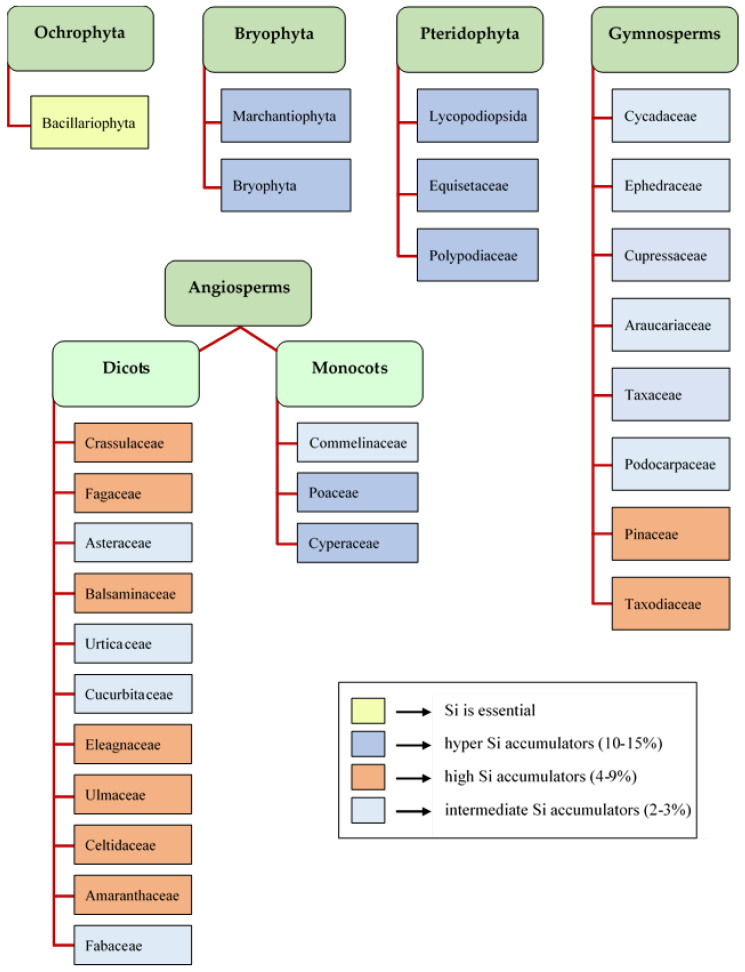
Distribution of different levels of Si accumulation in the plant kingdom [2,17,18,19,20].

Si uptake of plant species differs greatly resulting in significant differences in silicon accumulation [2]. Terrestrial plants can be grouped according to their ability to accumulate silicon in their tissues [32]. The silica content of species has been experienced ranging between 0.1 and 15% (dry weight basis). There are hyperaccumulators (10–15% dry weight) such as *Bryophyta* species [33], *Lycopsida* and *Equisetopsida* species of *Pterydophytae*, *Balsaminaceae*, *Cyperaceae*, and *Poaceae* of *Angiospermatophytae* of higher plants. Species among intermediate Si-accumulators (1–3% dry weight) are sunflower (*Helianthus annuus* L.), *Cucurbitales*, *Urticales*, and *Commelinaceae* species [34]. As research has proved, the typical silicon accumulating crops are mainly monocots, such as rice (*Oryza* spp.), sugarcane (*Saccharum officinarum* L.), wheat (*Triticum* spp.), etc., and most of the dicotyledons are nonaccumulators (<1% dry weight) (Figure 1). But some dicots, such as soybean (*Glycine max* (L.) Merrill), sugar beet (*Beta vulgaris* L.), are silicon accumulators [35]. Cucumber (*Cucumis sativus* L.) and tomato (*Solanum lycopersicum* L.) are in a special situation since the concentration of silicon in the root cells is higher than in the surrounding soil solution, despite the fact that they are known as not silicon accumulators now [36,37].

Ma and Takahashi [1] found that Si-rich species have generally low calcium concentrations. Based on plants’ Si concentration and [Si]/[Ca] ratio they classified plants into three groups such as “Accumulators” with Si concentration over 1% and a [Si]/[Ca] ratio >1, “Excluders” with Si concentration below 0.5% and a [Si]/[Ca] ratio <0.5, and “Intermediates” plants do not meet these criteria.

There is a huge literature on Si-uptake methods in different plants and the different ability of accumulation of silicon entails a different way of Si uptake [38], but the molecular mechanisms of the uptake and transport are not perfectly understood. Some species passively transport Si (through transpiration from the soils to shoots) while others also actively transport silicon and deposit it in leaf tissues (mainly in epidermis cells) at high concentrations [39]. There are plants (e.g., tomato, cucumber, beans) that exclude Si from uptake [40].

Takahashi et al. [41] reported that there are passive silicon uptake species such as oats (*Avena sativa* L.), cucumber (*Cucumis sativus* L.), melon (*Cucumis melo* L.), strawberry (*Fragaria vesca* L.), and soybean (*Glycine max* (L.) Merrill). Silicon uptake for cucumber needs clarification since it is defined as passive by Takahashi et al. [41], and Mitani and Ma [36], while active by Liang et al. [6], and Wang et al. [42]. Faisal et al. [39] proved that it is the passive way to take Si up, by cucumber, under a given transpiration rate provided by under changing humidity content and flowing air in a factorial experiment, which can significantly increase silicon accumulation in the leaf over four days. Deshmukh et al. [43] proved that soybean, Chiba et al. [44] that barley, Mitani et al. [45] that maize, Mitani et al. [46] that pumpkin cultivars, Vivancos et al. [47] that horsetail (*Equisetum arvense*), and Sun et al. [48] that tomato plants are active silicon transporters.

Liang et al. [49] reported that passive and active Si uptake mechanisms co-existed in the tested plants (*Oryza sativa*, *Zea mays*, *Helianthus annuus*, *Benincase hispida*) in their research.

Different Si transporters assure active processes. Influx and efflux transporters are responsible for Si-uptake by roots and towards xylem (xylem loading). Lsi1 (influx transporter) assures Si uptake from the soil solution into the root cells’ symplast, while the Lsi2 is an efflux transporter from the symplast to the apoplast. Lsi6 (influx transporter) is responsible for the transport of silicic acid from the xylem into xylem parenchyma cells (xylem unloading) [50]. Finally, Si is deposited into the epidermis cell walls as hydrated amorphous polymer (opal) forming silica–cuticle double layers and deposited also in specific shoot cells [51,52], and specialized epidermis cells called phytoliths. Species such as rice, wheat, ryegrass, barley, maize, banana, and *Cyperaceae* use active transport to uptake silicon [53,54,55]. Rice differs from the other plants in the role of root hairs in silicon uptake, which is lower compared to lateral roots [56].

Plants transport Si in various ways. They take up Si in the form of the monomeric, uncharged molecule of monosilicic acid (H_4_ SiO_4_) [57], which is then transported via the xylem wherein losing water through the transpiration causes increasing concentration. The mechanisms of protection of the condensed solution against polymerization are not yet fully understood. The polymerization takes place at the end of the transport and a transformation into colloidal silicic acid (polysilicic acid), then into silica gel (SiO_2_ × nH_2_O) at the end of the process [58]. Kumar et al. [59] reported a basic protein Siliplant1, which plays role in the polymerization of silicic acid and accumulation of Si in the cell walls. Plant species differ in the deposited silicon forms, their ratio, and Si accumulator cell types.

## 5. Phytoliths

Term phytolith is a composite of phyto (plant) and lithos (stone), both words of Greek origin. The silicon dioxide required for the formation of phytolith is absorbed in the form of ortho- or mono-silicic acid by the plant through the root under appropriate pH (pH 2–9) conditions [60]. It depends on the species and whether it is either a passive or an active transport. 

Phytolith is an isotropic material accumulating only in tissues of alive plants performing metabolism, which is hydrated quartz (SiO_2_ × nH_2_O) but contains very small quantities of other elements as well. According to Bartoli and Wilding [61], it is trace quantities of Mg, Ca, Na, K, Mn, Fe, Al, and organic carbon that phytoliths contain. A body of papers discussed that aluminum (Al) is co-deposited with silica in phytolith [62], however, they are enriched in terrigenous elements (Al, Sc, Ti, V, Cs, Fe, etc.), but hardly contain inorganic elements (K, Ca, Mg, Mn, Cl, Br). Recent findings suggest that the chemical composition of phytolith depends on the method of phytolith extraction. 

There are three methods of phytolith extraction applied the most such as dry ashing, acid digestion, and acid digestion followed by incineration. Using these procedures when examining barley organs (stem, leaf, awns) it was stated that dry ashing and acid digestion followed by incineration method proved to be effective for phytolith extraction, but dry ashing led to lower elements in the extracted phytolith than acid digestion followed by the incineration method [63]. 

**Figure 2:**
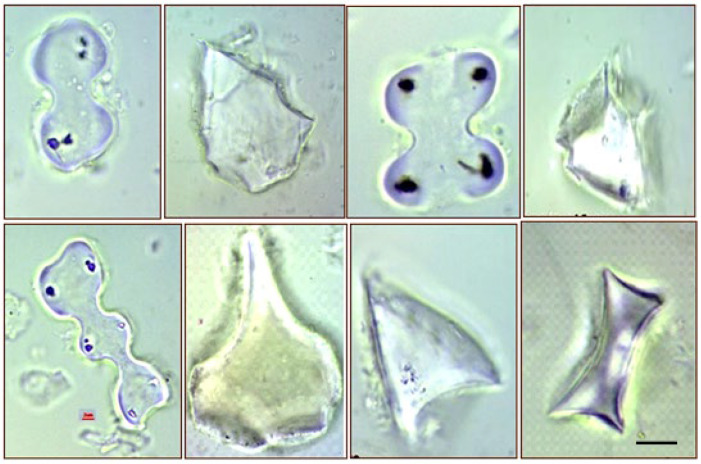
Some phytolith morphotypes of *Arundo donax* L. ecotypes (Photo: Dr. Szilvia Kovács). Upper line left to right: bilobate, blocky, cross, pyramidal, bottom line left to right: polylobate, bulliform, scutiform, rondel. *Scale bar*: 5 µm).

Phytoliths could be 5–200 μm, but in most species are 10–30 μm in size. Ophthalmic particles that are well visible, clearly recognizable and determinable with a light microscope (Figure 2). They vary in color (pink, yellow, and gray translucent) and vary in weight from 1.5 to 2.4 g cm^−3^. Although it is formed in a living plant, it is released after the plant has died and is transported to a subsequent storage medium (sediment, soil) [60].

It is important to emphasize that the ability of plants to produce phytoliths can vary at the cellular, tissue, organ, and organism (individual) levels. This is due to the fact that the formation of phytolith is significantly influenced by the different climates, the different chemical and physical properties of the soil, the age, and taxonomy of the plant. Phytolith formation in the plants is observed at the cellular level in three distinct locations: deposited on the cell wall, inside the cell, and in the intercellular passages of the cortex [64]. The hydrated, amorphous silica may also be deposited in the cell walls, inside the cells, and in the intercellular spaces or external layers. In this form, Si is immobile and redistribution is not possible [65].

There are differences in the accumulation of phytoliths at both organ and tissue levels. Typically, they occur in the primary cortex of the root, in the epidermis of the leaf, in the bracts in the inflorescences of the lawns, in the epidermal cells of shoots, or in the pericarp of the fruit. Sangster [66], examining the roots of *Sorghum bicolor* (L.) Moench and *Sorghastrum nutans* (L.) Nash, found that Si was deposited on the inner tangential wall of the tertiary phase endodermal cells. Comparing his results with other *Poaceae* species, he found that differences in the distribution of Si in the root is depending on the phylogenetic status of the species within the genus, not on the anatomy of the root. Examining wheat leaves, Hodson and Sangster [67] found that silicification is stronger in the lower epidermis of the young leaves and the upper epidermis of the older leaves (adaxial epidermis).

### Areas of Practical Use for Phytoliths

Unique size and shape, with precise morphological description, and often species- or taxon-specific opal grains, are well suited for palaeobotanical, palaeoecological, and archeological reconstructions. Lisztes-Szabó et al. [68] pointed out that phytoliths may be applicable to reveal intraspecific variance of frequency and size, within the phytolith assemblage of the same species (e.g., *Poa pratensis* L.). By their examination, we can infer the species composition of earlier vegetation that has disappeared today, the climatic conditions, the conditions of sediment formation, and the conditions of early anthropogenic activity (lifestyle, food, crop production) [60,69,70].

In plants, the beneficial effects of silicon are emerging based on both physiological and mechanical mechanisms due to the presence of phytoliths. Through these mechanisms it alleviates Mn, Cd, As, Al, and Zn toxicity, reduces the excess absorption of nutrients (P, N), improves K, P, Ca intake, alleviates effects of different abiotic and biotic stresses (P deficiency, salt, drought, pathogens, and insects), increases tolerance to strong wind and rain [17]. Silicon in plants deposited on the tissue surface, thickens the epidermal layer, which leads to increased rigidity of plant tissues and becomes a mechanical barrier to pests [71]. The rigid silica structure of phytoliths provides structural support to the plants and reduces the digestibility of grasses for small herbivores, although it cannot save the plants from large vertebrate herbivores [72]. Also, it reduces the digestibility of the plant cells making them less susceptible to enzymatic degradation by fungal pathogens. Cherif et al. [73] proved that silicon is a signal to induce the production of phytoalexin.

Recent research has shown that phytolith occluded carbon (PhytOC) is stable in the soil for thousands of years and can accumulate in soils, therefore, it offers an opportunity to enhance terrestrial carbon sequestration [74]. Some of the most important crops (for example, barley, maize, rice, sorghum, sugarcane, and wheat) produce PhytOC [75].

## 6. Effects and Possibilities of Silicon Fertilization

Researchers found long time intensive cultivation, the application of chemical fertilizers can cause decreasing in the available silicon content of soil and could be a real limiting factor to getting high yields, especially for silicon-accumulating crops [76,77]. In general, tropical and subtropical soils are scarce in plant-available silicon [78]. Si is, therefore, now a so-called “agronomically essential element”; applying silicon-containing fertilizers has become more frequent, and many farmers have already included it in their crop fertility programs. Most silicon fertilizers may be used in certified organic production systems. 

**Figure 3:**
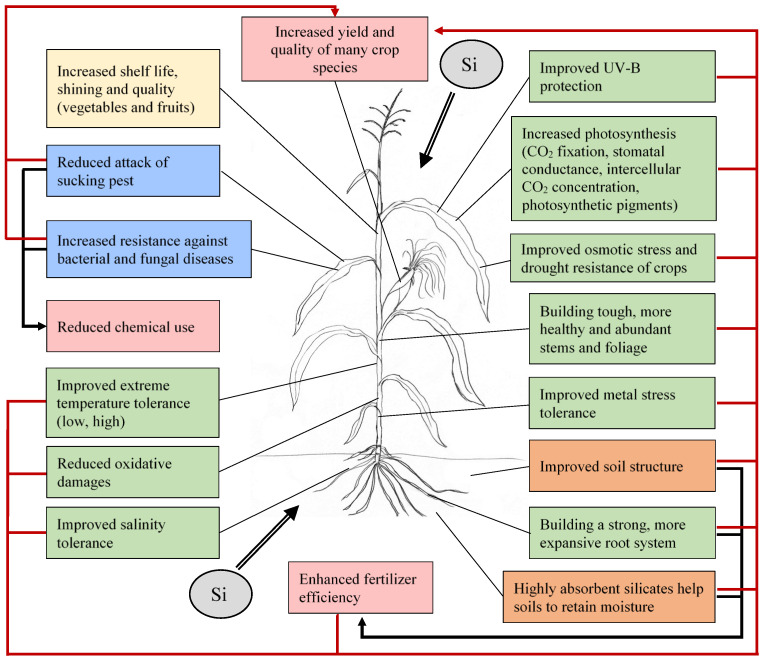
Effects of silicon application in the crop production space.

Research was carried out to clarify the beneficial effects of the application of silicon-containing fertilizers on plant growth and development. The use of silicon fertilizers improves soil structure and using highly absorbent silicates increases soil moisture retention. The improved soil structure and water-holding capacity, as well as a more developed root system, also improve the efficiency of fertilization. The positive effects of silicon application on vegetation development and vigor can be observed at several points: improved abiotic stress tolerance (UV-B radiation, osmotic stress, metal and heavy metal stress, extreme temperature stress, oxidative stress, salt, and salinity stress), and biotic stress tolerance. The complex system of the beneficial effects and their interactions improve plant health and increase yield. Si addition has positive effects on the photosynthetic characteristics of plants, like higher values of photosynthetic pigment content, photosynthetic rate, stomatal conductance, and intercellular CO_2_ concentration can be observed [79,80,81,82]. The precise mechanisms that regulate stomatal closure and the CO_2_ signalling pathway that is part of it, and the processes that regulate it, are not fully understood. Related research concerns the function of the CO_2_ and abscisic acid (ABA) sensors in guard cells, the function, role, and the activation mechanism of SLAC1 (slow anion channel in the membrane of stomatal cells) and the guard cell-specific promoters, expression of genes encoding proteins involved in the closure regulation process, e.g. the RHC1 gene. [83,84,85,86]. Silicon application also increases the shelf life, shining, and quality in the case of vegetables, fruits, and flowers [87]. The application of silicon fertilizers has positive environmental impacts due to the reduced use of plant protection chemicals (Figure 3). 

**Figure 4:**
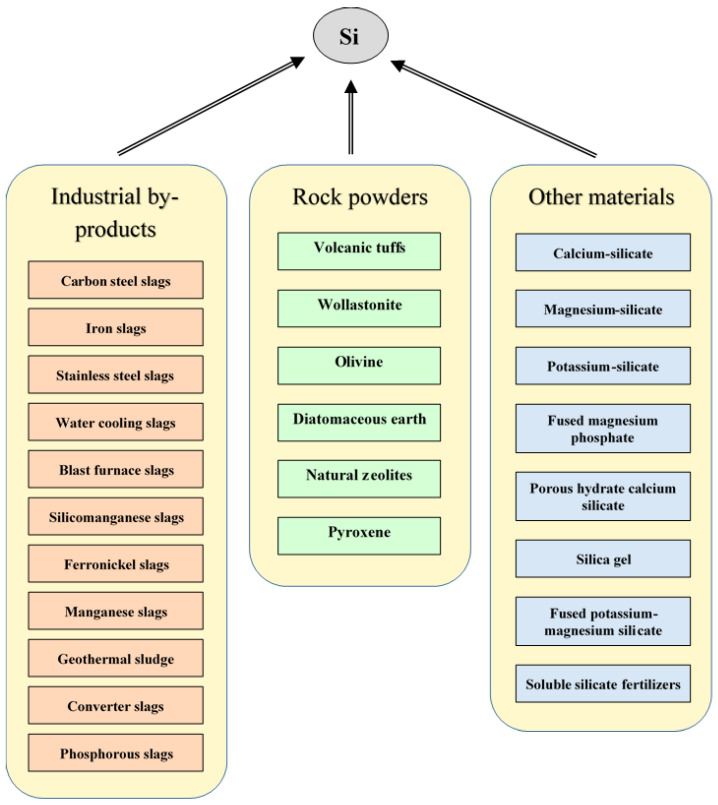
The silicon-containing materials, originated from various organic and inorganic sources, usable as fertilizers.

Silicon fertilizers come from various organic and inorganic sources, such as those derived from volcanic tuff, industrial by-products, rock powders, and other materials (Figure 4).

The silicon content available to plants varies in different forms of Si fertilizer [88]. Pereira et al. [89] found that some extraction methods overestimate the amount of silicon available to the plant from the fertilizers. In the opinion of Buck et al. [90], it is possible to estimate total silicon content from a potential Si fertilizer source, but this does not reflect the amount available for uptake by the root system of plants. They found that the difference can be large and Na_2_CO_3_ + NH_4_NO_3_ is suggested as extractor for solid fertilizers and HCl + HF for liquid fertilizers. In liquid fertilizers, all of the silicon content is almost completely soluble.

Harley and Gilkes [91] investigated the effectiveness of different rock dust applied to the soil in supplying silicon to plants. They stated that the results are influenced by a number of factors, such as climatic conditions, soil solution composition, pH, redox processes, and the rhizosphere activities. Organic acids released into the rhizosphere may greatly enhance the weathering rate of silicate minerals and the rate of silicon uptake by plants. 

Makabe-Sasaki et al. [92] reported that chemical properties (Si adsorption capacity, contents of Si adsorbents (acid oxalate-extractable iron and manganese) and the pH of the soil has a great effect on the efficiency of slag silicate fertilizer application in Japanese rice fields. The research has shown that methane (CH_4_) emission can be significantly reduced in rice fields using slag silicate fertilizer [8,93]. The extent of reduction depended on the iron oxide content of the slag, with higher iron dioxide content resulting in lower methane emissions. Song et al. [94] found a positive correlation between the silicate fertilizer application and phytolith production flux and carbon trapped in phytoliths (PhytOC). Since PhytOC accumulates and is stable in soil [65], silicate fertilizers can be an effective tool in improving atmospheric CO_2_ sequestration. 

Bocharnikova et al. [95] examined the efficiency of various silicon fertilizers (solution of monosilicic acid, liquid silicon–humic fertilizer, diatomite, and calcium silicate) on rice (*Oryza sativa* L.), wheat (*Triticum aestivum* L.), corn (*Zea mays* L.), barley (*Hordeum vulgare* L.), and cucumber (*Cucumis sativus* L.) crops in field tests on different soil types. All types of silicon fertilizers substantially increased crop yield on all soil varieties. One side-effect was found that silicon fertilizers can support and increase the transformation of the unavailable phosphorous content of the soils into available forms for plants. As their results of field experiments showed, applying liquid or solid silicon fertilizers led to the replacement of the phosphate anion by the silicate anion from calcium, aluminum, and iron phosphates. In advance, preventing the strong fixation of phosphate anions in the soil, silicon amendments application increased the efficiency of phosphorous fertilizers by 30–50%. They suggested using this effect on contaminated soils where dephosphating is needed. Although Agostinho et al. [96] experienced an improvement in biomass (42%) and tiller production (25%) for rice as an effect of foliar Si fertilization, they found that the most effective way for increasing Si uptake by plants is to apply Si-rich materials to the soil.

Cook [97] found in his investigation testing the effect of available silicate slags on the crops, that the application of different silicate slags increased the phosphorous uptake and the yield of barley. He did not find evidence of the increased phosphorous uptake from the applied superphosphate fertilizer. The silicate slags have an effect mainly on the mobilization of natural phosphorous content of the soil (in his opinion).

The use of different industry originated slags as silicon fertilizers is relatively well documented and investigated in different crops but particularly so in rice production. Ning et al. [77] found increased growth and disease resistance as a result of the application of slag-based silicon fertilizer on rice crops, but the results varied depending on the slag source. Steel and iron slags were involved in their research under greenhouse conditions. Both slags were beneficial to the growth and yield of rice, but steel slag showed stronger effect on increasing the disease resistance. They suggested that the differences were caused by the varied presence and availability of silicon and other nutrients in the slags. The industry uses raw materials originated from different sources or locations and this variation is reflected in the different quality of the slags. 

Datnoff and Rodrigues [5] investigated the possibilities of silicon fertilization. They found long-time residual activity in the soil after the application of some silicon fertilizers. According to these results, they suggested significantly lower subsequent application rates after initial silicon fertilization. They also proposed the possibilities of using alternative silicon sources on fields, such as rice hulls or straw, since silicate slags have a high price. More studies were conducted aimed to investigate the effect of silicon fertilization on plants. Most of them report beneficial effects of application on plant growth, yield components, yield, and plant health [98]. In wheat, foliar spray of silicon fertilizers had significant effect on the yield and above-ground biomass production in Pakistan on silty loam soil [99]. 

Neu et al. [100] in their research conducted in Germany found that silica fertilization increased the aboveground biomass production and nutrient (especially nitrogen) use efficiency of wheat and grain yield also increased at medium Si supply level. The application of silicon fertilizers on alluvial soils increased the total chlorophyll content and photosynthetic ability of maize leaves [101,102].

Shwethakumari and Nagabovanalli [103] found that silicon fertilization had been beneficial to soybean plants in India. They applied silicic acid (H_4_SiO_4_) in 2% concentration as foliar spraying on soybean plants. The treatment resulted in significant increase of both the growth and yield of soybean. 

In table grape plantations, silicate fertilizer application (600 kg SiO_2_ ha^−1^) increased both the yield and quality in China. Not only the yield, but the cluster weight, berry weight, ad berry size (i.e., length and width) were significantly increased by application of silicon fertilizer in both involved cultivars. The total soluble solids and berry firmness were improved and suggested prolonged shelf-life as a result of the treatment [104]. 

Research results show that liquid silicon fertilizer amendments applied as foliar treatment can increase the nutrient value of crops such as silage crops. The silages made from silicon fertilized plants had a higher nutritional value and feeding dairy cows with silages made from Si-fertilized plants improved their milk productivity and the milk quality parameters (fat, protein, somatic cells count) [105].

Potassium silicate provides an excellent source of soluble silicon for plants and provides also potassium for plants [106]. Potassium silicate containing steel industry slags are in testing as slow-release potassium fertilizers. Muljani et al. [107] found that the effect of potassium silicate slags depends on the type of raw material and potassium salts used. Wu and Liu [108] suggested that the slow-release potassium silicate fertilizer can be useful in agriculture, due to its excellent water retention capacity (85 times its weight) and the slow release of potassium and silicon nutrients. In Chinese cabbage (*Brassica pekinensis* Rupy cv. Kekkyu) culture Yao et al. [109] found that the release of organic acids such as citric acid by the roots system could effectively accelerate the solubilization of sparingly soluble K in slow-release potassium silicate fertilizer. 

Cucurbits are silicon accumulator plants [1]. Gorecki and Danielski-Busch [110] studied to test the reaction of cucumber to silicon fertilization. They applied slow-release Ca- and NH_4_-silicates and other silicates as Si source on peat substrates. Ca- and NH_4_-silicates increased the accumulated Si content of the leaves and fruits and improved the resistance to biotic (diseases) and abiotic (drought) stresses. The yield was also higher, but mainly as a result of the higher number of fruits, not through the average fruit weight. The effect of the other silicates was unambiguous (even as high as 4 g liter^−1^). Substrate Ca- and NH_4_-silicates concentration was beneficial for cucumber plants. 

Sugarcane is one of the most sensitive crops for silicon deficiency. Elawad et al. [111] found, that application of silicate materials in sugarcane increased leaf chlorophyll content (by 78 and 65%) and decreased leaf freckling (by 46 and 41%) in both plant crops and ratoon crops. They did not find significant differences among the silicate materials. Silicate materials also resulted in increased soil pH, soil silicon, phosphorous, calcium, and magnesium content. 

The use of Ca-silicate containing materials can be one solution. Calcium silicate slag application increased the cane yield of plant and ratoon sugarcane by 39% and sugar yield by 50% in the Everglades Agricultural Area (USA), but the magnesium concentration in the leaf was critical [112]. He examines the question concerning the silicon-magnesium antagonism and suggests additional magnesium fertilization in case of calcium silicate slag application on the fields (although this may depend on the soil properties as well).

Bokhtiar et al. [113] tested the effect of growing Ca-silicate amendment levels to yield and gas exchange parameters of sugarcane. Silicate-amended treatments significantly increased maximum dry matter and cane yield by 15–77% depending on the soil. Increasing silicate application significantly decreased the iron, copper, zinc, and manganese contents in leaf tissues and soil.

## 7. Silicon—A Valuable Tool for Regulating Biological and Abiological Stresses in Crop Production

The importance of silicon fertilization is increasing due to its potential mitigating soil nutrient depletion and biotic and abiotic environmental stresses. Liang et al. [6] stated in their study that Si is the only mineral element known effectively can moderate the effects of abiotic stresses (salinity, drought, flooding, freezing, high temperature, ultraviolet radiation, and mineral nutrient deficiency or toxicity). Zargar et al. [114] considered silicon plays an advantageous role in defense against both biotic and abiotic stresses in plants. They concluded that silicon should use regularly as other fertilizers (especially in Si accumulator crops such as most cereals and monocots and furthermore in some dicot plant species as well). 

Silicon could be a valuable tool for maintaining sustainable agriculture, using environmentally friendly strategies for the management of plant diseases and pests [115]. Its benefits are well-demonstrated in the case of silicon-accumulating crops exposed to environmental stresses on soils with low silicon content [17].

### 7.1. Abiotic Stresses

#### 7.1.1. Function of Silicon in Mitigating Plant Nutrient Imbalance and Improving Nutrient Use Efficiency

Researchers studied the effect of silicon application in the alleviation of P imbalance stress in a variety of plant species. These studies have shown that Si supplementation has a positive effect on P-imbalance stress in a variety of plant species, through a variety of biochemical and physiological processes. P-deficiency stress can be alleviated by increasing P mobility, lowering exchangeable Al^3+^ in acid soils, boosting malate and citrate exudation, upregulating P transporter genes, and enhancing internal P consumption by decreasing Fe and Mn uptake. Exogenous Si’s beneficial effects on excess-P stress can be related to the formation of apoplastic barriers resulting from Si deposition in root cortex cells, as well as the downregulation of P transporter genes [116].

In maize and wheat plants, silicon amendments improved leaf chlorophyll index, N-uptake and as a result, agronomic efficiency of nitrogen fertilization [117], due to improved shoot and root development and finally greater grain yields (an increase of 5.2 and 7.6 percent, respectively).

In rice plants long-term S stress resulted in a higher accumulation of Si in the shoots, which balanced the source-sink metabolite homeostasis and compensated for the lack of shoot S. Silicon effectively reduced stress levels, as seen by the lower accumulation of stress phytohormones, allowing plants to grow and develop despite S deprivation [118].

Hosseini et al. [119] revealed that Mg-deficient maize plants treated with silicon nutrition maintained their growth and increased the chlorophyll level and soluble sugar content as an effect of Si nutrition by regulating plant primary metabolite and hormonal changes.

Silicon treatment helped the fast recovery of Zn-deficient cucumber plants grown in hydroponics, suggesting that Si could be utilized to prepare plants to cope with a future stress situation, such as nutrient deficiency [120].

#### 7.1.2. Function of Silicon in Mitigating Metal and Heavy Metal Stress

The rising problem is the contamination of soils with trace elements and heavy metals, causing a serious environmental issue and a factor of food contamination [121]. It can cause physiological disturbances (reduced biomass production, photosynthesis inhibition nutrient uptake problems) in plants. Silicon applications may take an effect at different levels, either in the plant or in the soil. Reduced metal ions in soil substrate, toxic metal co-precipitation, metal-transport related gene regulation, chelation, antioxidant stimulation, metal ion compartmentation, and structural alterations in plants are some of the basic mechanisms involved in Si-mediated heavy metal stress tolerance [122]. There is a vast body of literature discussing metal toxicity to be reduced by silica. In soils, silicon may reduce Al, Zn, Mn, Cd, As, and Fe toxicity and can make soil phosphorus content more accessible [17]. Sarwar et al. [123] found that silicon may reduce toxicity symptoms of metals such as cadmium. Zaman et al. [124] obtained similar results and noted that the effectiveness of silicate compounds in reducing Cd toxicity varied with the kind of chemicals, doses and time of foliar applications.

Wu et al. [125] examined the effect of silicon fertilization on arsenic (As) uptake in different rice plant genotypes. They found that Si significantly increased the straw biomass without increasing root biomass. The Si fertilization reduced total arsenic concentration in shoot and root, so it is an efficient tool to reduce As contamination of rice grown on As contaminated soils. Ali et al. [126] concluded, that Si supplementation is beneficial in relation to different micronutrient and heavy metal stress via reducing oxidative stress and reducing intercellular availability of toxic elements with co-deposition of these elements in the cell wall together with Si.

Song et al. [127] reported that silica was to mitigate the negative effects of Zn-stress on photosynthesis. They applied in hydroponics experiments on rice and justified that a total of 15 mM of extra Si can manage to mitigate the malfunctioning of photosynthesis under high Zn-stress. As a result of Si feeding, the damaged chloroplast ultrastructure was restored (thylakoid regeneration, increasing starch size and number). 

#### 7.1.3. Function of Silicon in Mitigating the Negative Effect of Heat and Drought Stress

Droughts have more and more significance in crop production due to climate change and limited water resources (especially in certain regions of the world). Bocharnikova et al. [95] conducted research aiming to determine the effect of silicon fertilizer on the drought resistance of barley. They applied amorphous silicon dioxide, calcium silicate, and monosilicic acid solution (Si concentration of 150 mg L^−1^) in climate chambers. Biomass production of barley increased by 10–53% as a result of applying silicon-containing compounds, and the plants showed more resistance to drought stress. Other experiments proved the positive effect of silicon application on maintaining the water potential, reducing the negative impact of drought stress and increasing the yield and biomass production of wheat [128,129,130,131]. On the contrary, in case of wheat landraces, during drought stress, Si had no substantial effect on growth, and during osmotic stress, Si only modestly improved growth in high Si accumulators [132]. The wheat landraces differed significantly and reliably in their Si accumulation. Drought raised Si content in all genotypes, whereas osmotic stress lowered it.

Kutasy et al. [133] based on their experimental results established, that Si can alleviate the drought stress of oat by improving the photosynthesis rate (16.8–149.3%) and water use efficiency, adjusting the chlorophyll content and stomatal conductance, and regulating transpiration, and as a result, the yield increased by 10.2%. Great variation was found in response to the foliar Si fertilization among the examined winter oat varieties.

Silicon application increased the leaf area, chlorophyll content, enzymatic antioxidant activity [134], improved the photosynthetic rate, lowered the transpiration rate of drought-stressed maize plants, and as a result increased the dry matter production and grain yield [101]. 

Research has demonstrated that potatoes also react well to silicon fertilization. In an experiment aimed the effects of silicon on potato (*Solanum tuberosum* L.) plants under drought stress in Brazil. The results show that silicon application and water deficit resulted in the greatest Si concentration in potato leaves, and the silicon treatment increased the average tuber weight, dry tuber weight, and tuber yield [135]. 

Gugala et al. [136] conducted research on the effects of silicon application on rapeseed development, overwintering and yield. They stated the foliar application of silicon-containing growth stimulator increased the yield from 1.7 to 17% depending on the variety and the treatment also had positive effect on the hardiness of the rapeseed plants. Even non-accumulator species such as oilseed rape collect silicon under certain conditions, improving water uptake during drought stress [137].

Drought and UV-B stress have the same effect on Si-treated plants. Si boosts photosynthetic rate and chlorophyll content in plants subjected to high UV-B radiation and drought, lowers catalase and superoxide dismutase activity, and lowers malondialdehyde content [138]. As a result, plants have fewer reactive oxygen species.

#### 7.1.4. Function of Silicon in Mitigating the Negative Effect of Salt Stress

One of the most important abiotic stressors affecting development and production is salinity. Plants are stressed by oxidative, osmotic, and ionic stressors when there is a high concentration of salt in the environment. Salinity impacts soil, groundwater, and agricultural production in extreme cases. In a variety of plants, Si reduces salt stress [139]. Photosynthesis, detoxification of toxic reactive oxygen species via antioxidants and non-antioxidants, and correct nutrition management are all involved in Si-mediated stress reduction [140]. The foliar application of silicon led to improvements in concentrations of chlorophyll a and b and mineral nutrients, water status, and fruit yield of sweet pepper plants. Furthermore, lipid peroxidation, electrolyte leakage, levels of superoxide, and hydrogen peroxide were decreased with silicon treatments [141]. The effects of Si on plant salt tolerance differ depending on the species and cultivars. This could be related to the Si uptake capabilities of plants. Furthermore, the concentration of Si, the duration and intensity of stress, the cultivation methods used for experimental materials (hydroponics and soil culture), Si application methods (foliar and root application) and forms (silica ions, stabilized silicic acid, and silica nanoparticles), all of which influence the regulatory effects of Si on salt tolerance in plants [142].

#### 7.1.5. Function of Silicon in Carbon Sequestration

It has been known for a long time, that silica can replace carbon in biomass, that is, silica accumulation reduces carbon content in species of silica-accumulators (mainly in *Poaceae*). This is particularly true for cell walls (cellulose and lignin) and phenols [99]. Schaller et al. [143] using an FTIR spectrometer and stated that the proportion of other compounds containing carbon (fat, wax, lipids, organic acids) was decreased by silica accumulation. Furthermore, an examination of pigmented red and non-pigmented brown rice varieties pointed out that species differently influence the quality of plant carbon in the leaves. Carbon quality has a basic influence on the decomposability of leaves, effecting the whole grassland dominated ecosystem.

### 7.2. Biotic Stresses

#### 7.2.1. Disease Resistance

Silicon plays an important role in the disease and pest resistance of many plant species. In recent years several scientific reports can be found connecting to the role of silicon in the disease resistance of plants [11,77,144]. Datnoff and Rodrigues [5] suggested silicon fertilization as an effective and cost-saving tool in the control of several plant diseases. They wrote there was not in doubt its ability to control the diseases efficiently, and besides this, it is an environmentally friendly solution, as well. The beneficial effects of silicon amendments in plants’ self-defending ability against many pathogens are widely accepted [17]. Buck et al. [145] stated according to their research in rice, that the foliar application of liquid silicon fertilizer is a practical solution for increasing the resistance and reducing plant diseases at low cost. Ning et al. [77] found that slag-based silicon fertilizers have beneficial effects on the growth and disease resistance of rice, but the effect depended on the sources of the slag.

According to research work conducted in rice [146], silicon fertilization can be a good tool in disease control and improve yields of rice and other related crops. In their experiment silicon reduces susceptibility in rice to fungal diseases significantly. Their opinion that silicon fertilization can control rice diseases to the same general degree as a fungicide application. The beneficial effect of silicon amendment expressed on those soils where the natural available Si content is below the optimum level. They suggested using silicon amendment in integrated pest management programs, in particular in such crops in which the positive effect of silicon fertilization is proved. Silicon treatment increased yields more effectively than fungicides alone. Therefore fungicides might be eliminated altogether (or the number of fungicides applications in a growing season can be reduced significantly). The reduced application of chemicals has positive environmental benefits and causes decreasing in costs, as well. Ca-silicate application as foliar spray decreased the damage caused by rice blast disease.

Silicon-containing growth stimulator (94 g L^−1^ Si) treatment significantly reduced the deoxynivalenol (DON) and zearelon (UAE) content of the maize grains [147]. Si application reduced the severity and relative fungal growth of Red Crown Rot (RCR) caused by *Calonectria ilicicola* in the roots of soybean plants. The amount of Si significantly correlated with the severity of RCR. Si-treated plants also had better water and nitrogen uptake, as well as root and shoot growth. With increasing Si concentration (0.0–3.0 g Na_2_SiO_3_ kg^−1^ soil), aboveground biomass and seed yield at harvest increased, but higher (6.0 g Na_2_SiO_3_ kg^−1^ soil) concentration caused the yield to decrease [148].

Njenga et al. [149] determined the potential effect of silicon fertilization on the resistance of cassava (*Manihot esculenta* Crantz) to bacterial blight (CBB). The pathogen bacterium is *Xanthomonas axonopodis pv. manihotis* (Xam). They studied the plants’ physiological and biochemical mechanisms in the development of resistance induced by the silicon amendment. They also involved two sensible cultivars to CBB, TME14 and TMS60444. Silicon application significantly increased the resistance of cassava plants and reduced CBB infection in all of the eight cultivars tested.

#### 7.2.2. Pest Resistance

Silicon has long been associated with increased ability to withstand attack by pests in a wide range of crops, in different ways [150]. Si can reduce the damages caused by herbivorous animals, such as insect pests [151] or wild rabbits, furthermore grazing animals do not prefer silicon fertilized grasses [72,152]. Although research mainly focused on high-Si accumulators, the results suggest that silicon accumulators and non-accumulators, as well as monocots and eudicots, all have similar Si defense mechanisms against insects [153]. The mechanism of the resistance is thought to be the amorphous silica that builds mechanical barriers including accumulation of lignin, phenolic compounds, and phytoalexins in the plants [2,154,155,156].

In horticulture, the application of silicon fertilizers also can be beneficial. Parella et al. [157] tested the effect of a range of potassium silicate fertilizer doses on the *Chrysanthemum* plants grown in pots. At 200 ppm and higher, they observed a significant reduction in leafminers’ damage. They concluded that the silicon may increase the chrysanthemum’s ability to withstand the attack of leafminers.

In tomato plants, the application of calcium silicate fertilizer increased the resistance to common blossom thrips (*Frankliniella schultzei* Trybom) pest. The result depended on the number of applications. More applications increased the mortality of nymphs and reduced the damage by the insect on tomato leaves (especially when it was applied together with organic mineral fertilizer) [158]. 

Hou and Han [159] studied the effect of the silicon amendment on resistance against early instar larvae (*Chilo suppressalis* Walker) in a susceptible (Shanyou63) and a moderately resistant (Yanfeng47) rice cultivar. They found silicon treatment significantly reduced the damage caused by the larvae (borer penetration, weight gain and stem damage). Leaf penetration duration and larval development time were prolonged. In conclusion, silicon fertilization may be a useful tool in integrated pest management (especially in susceptible genotypes). 

Silicon application induced the resistance in rice plants to the rice stalk stink bug (*Tibraca limbativentris* Stal). Silicon fertilization increases the silicon content and chlorophyll content of plants, in consequence, the resistance of rice plants, either it was sprayed on the plants or applied on the soil. Si treatment increases the chlorophyll content of plants resulted in lower percentage of damaged stems in the rice cultivars [160]. 

Jeer et al. [161] also found that silicon application reduced pest damages in rice. They tested the effect of rice husk ash and imidazole, alone and in combination, against damage by yellow stem borer (*Scirpophaga incertulas*, YSB) in five rice cultivars. The results showed that the treatments increased the deposited Si content of the plants, and decreased the damage caused by the pest. The yield increased significantly, too.

The effect of silicon amendment on the damage of root-feeding herbivores, especially rice water weevil (*Lissorhoptrus oryzophilus* Kuschel) was tested by Villegas et al. [162] under field conditions in rice. They found calcium silicate slag reduced the density of weevil larvae but did not affect the density of whiteheads caused by stem borers. The weak effect of silicon against insect pests thought to be due to the fact that the older plants and leaves have higher silicon content than younger plants. In the young plant, silicon may not have accumulated at a sufficient level when rice water weevils attacked the plants.

Radkowski and Radkowska [163] found positive significant effect of foliar silicon fertilizer amendments on plant height, general condition and yield and quality of the seeds in timothy-grass (*Phleum pratense* L.). The plants treated with 74.9 g ha^−1^ silicon showed lower infestation rate with pathogens and pests than the control ones. 

## 8. Conclusions

In conclusion, as most major crops are silicon accumulators, applying silicon fertilizers have plenty of prosperous effects either in cases of unstressed or stressed plants. The beneficial effects of applying silicon are improving the soil structure and enhancing fertilizer efficiency. Silicon provides increased protection of plants under stressed conditions [164]. Silicon can help alleviate the negative effects of abiotic stress factors including drought, heat, cold, heavy metals, radiation, salt and lodging by different biological mechanisms such as altered photosynthetic rate, better water use efficiency, changes in stomatal conductance, increased water potential, structural reinforcement, alteration in mineral uptake and accumulation, alteration to phytohormone concentrations, and reductions in oxidative stress [165,166,167,168,169].

Plants must contend with several biotic stressors. The use of different silicon fertilizers can significantly improve the resistance of plants to biotic stresses. Plenty of studies have shown the adverse effect of silicon on pests and diseases. Silicon apparently can help to reduce the harmful effects of viruses, fungal, and bacterial pathogens and increase plant resistance to herbivores. The mechanisms of defense are quite similar whether pathogen infection or pests are involved. The researchers identified different defensive mechanisms such as physical defense by phytoliths increasing plant rigidity and physical toughness and conducting physical barrier to fungal penetration. Silicon deposition also reduces the plant digestibility to insects and mammalians. The silification of plant tissues is inducible, with more heavily attacked plants accumulating more silicon [2,165,170,171].

Silicon fertilizers in agriculture are still not commonly used, but their beneficial effects, especially in the case of silicon accumulating crops grown on poor quality soil, could be remarkable either in integrated, conventional, or organic farming. Silicon fertilization seems to be a tool to lessen environmental stresses and soil nutrient depletion and, subsequently, it could be an alternative for maintaining sustainable agriculture. Systematic molecular and genetic studies of Si-mediated mitigation of nutrient imbalance stress in plants should offer a theoretical background for Si fertilization in crop production. Further research is necessary to determine the optimal time and doses of silicon nutrition to specific crops grown in diverse edaphic and climatic circumstances in order to improve agronomic efficiency [172,173].

As silicon could be an effective tool controlling biotic stresses, it can help to reduce the number and rates of pesticides and save application costs while providing positive environmental effects [5]. The use of silicon accumulator crops can reduce the need for silicon supplementation, thereby reducing costs. Broadening the range of plant species or cultivars with improved silicon uptake and accumulation, and making greater use of the benefits associated with silicon in plants [102] could help to develop more sustainable cropping systems in the future and offer the potential to increase terrestrial carbon sequestration.

## Data Availability

Not applicable.
